# Fat Mass Follows a U-Shaped Distribution Based on Estradiol Levels in Postmenopausal Women

**DOI:** 10.3389/fendo.2018.00315

**Published:** 2018-07-02

**Authors:** Georgia Colleluori, Rui Chen, Nicola Napoli, Lina E. Aguirre, Clifford Qualls, Dennis T. Villareal, Reina Armamento-Villareal

**Affiliations:** ^1^Division of Endocrinology and Metabolism, Department of Internal Medicine, Baylor College of Medicine, Houston, TX, United States; ^2^Center for Translational Research on Inflammatory Diseases, Michael E. DeBakey VA Medical Center, Houston, TX, United States; ^3^Division of Endocrinology, University Campus Biomedico of Rome, Rome, Italy; ^4^Division of Endocrinology and Metabolism, Department of Internal Medicine, New Mexico VA Health Care System, Albuquerque, NM, United States; ^5^Division of Mathematics and Statistics, University of New Mexico School of Medicine, Albuquerque, NM, United States

**Keywords:** estradiol, obesity, adipocyte, adipose tissue, body composition, estrogen receptor, estrogen receptor alpha, estrogen receptor beta

## Abstract

**Objective:**

Estradiol (E2) regulates adipose tissue resulting in increased fat mass (FM) with declining E2. However, increased visceral fat and hyperestrogenemia are features of obese individuals. It is possible that adipocytes in obese individuals are less sensitive to E2 resulting in higher FM. Our objective is to identify the range of serum E2 for which postmenopausal women have the lowest FM and best body composition.

**Methods:**

Cross-sectional data from 252 community-dwelling postmenopausal women, 42–90 years old. Subjects were stratified into categories of E2 (pg/ml): (1) ≤10.5; (2) 10.6–13.9; (3) 14.0–17.4; and (4) ≥17.5. Body composition by dual-energy X-ray absorptiometry. Serum E2 by radioimmunoassay. Between-group comparisons by analysis of covariance.

**Results:**

E2 linearly increased with increasing body weight and body mass index (*r* = 0.15 and *p* = 0.01 for both), but not with total FM (kg) or % FM (*r* = 0.07, *p* = 0.34 and *r* = −0.04, *p* = 0.56, respectively). However, total FM (kg) followed a U-shaped distribution and was significantly lower in group 3 (27.6 ± 10.6), compared with groups 1: (34.6 ± 12.5), 2: (34.0 ± 12.4), and 4: (37.0 ± 10.6), *p* = 0.005. % FM was also lowest in group 3. While fat-free mass (FFM, kg) increased with increasing E2 (*p* < 0.001), % FFM was highest in group 3.

**Conclusion:**

In our population of postmenopausal women, FM followed a U-shaped distribution according to E2 levels. E2 between 14.0 and 17.4 pg/ml is associated with the best body composition, i.e., lowest total and % FM and highest % FFM. Given the role of E2 in regulating body fat, high FM at the high end of the E2 spectrum may suggest reduced E2 sensitivity in adipocytes among obese postmenopausal women.

**Clinical Trials:**

ClinicalTrials.gov identifier: NCT00146107.

## Introduction

Over one-third of US adults are obese (1). Considering the 10-fold rise in the worldwide incidence of obesity among children and adolescents during the past four decades, the number of obese adults could increase further in the coming years (2). High visceral fat mass (FM), common in obese individuals, is a risk factor for the development of cardiovascular disease, type 2 diabetes, and certain types of cancer, reason for which obesity is considered a major public health burden of the twenty-first century (1, 3). Research on the mediators of body fat deposition and distribution is thus crucial. Estrogen has been recognized as a regulator of FM (4–7), and in sufficient amount favors a “pear-shaped” fat distribution among women through promotion of both visceral lipolysis and subcutaneous adipogenesis (4, 8). Thus, the decreased lipid utilization and visceral FM accumulation in postmenopausal women is attributed to a severe reduction in estrogen levels (4–7). In rat adipocytes, estrogen depletion resulted in increased lipoprotein lipase and lipid deposition, while estradiol (E2) treatment reversed this process (9).

Although high E2 levels could be theorized as protective against developing obesity, it is interesting to note that obesity is classically characterized by relative hyperestrogenemia. It is in fact expected that increased aromatase activity in the expanded adipose tissue volume in obese individuals would result in high E2 levels (10). However, because these individuals remain obese, this observation may suggest impaired regulation of FM by E2 or reduced E2 sensitivity.

We thus hypothesize that total body fat varies according to E2 levels with individuals in the lower and higher end of the E2 spectrum having higher body fat. To the best of our knowledge, there are no reports describing the optimum E2 levels associated, primarily, with the lowest body fat and, secondarily, with the best body composition profile in postmenopausal women. The aim of this study is to determine the association of body fat and fat-free mass (FFM) with circulating E2 and to identify the range of serum E2 levels associated with the lowest body fat and the best body composition profile in postmenopausal women.

## Materials and Methods

### Study Population

This is a secondary analysis of cross-sectional data collected from otherwise healthy community-dwelling postmenopausal women participating in a study investigating the relationship between polymorphisms in the different CYP450 enzymes that metabolize estrogen and the effect on bone health (11, 12) or those undergoing a lifestyle intervention trial (13). These protocols were approved by the Washington University School of Medicine institutional review board. Both studies were conducted in accordance with the guidelines in the Declaration of Helsinki for the ethical treatment of human subjects. Participants were recruited using advertisement or direct mailing. All of the participants were provided written informed consent. Exclusion criteria include the use of medications that affect gonadal hormone levels such as estrogens and androgens, medications that affect bone metabolism such as glucocorticoids, anticonvulsants, bisphosphonates, selective estrogen receptor modulators (e.g., raloxifene and tamoxifen), aromatase inhibitors, as were medical conditions that affect bone metabolism such as hyperparathyroidism, chronic renal failure, Paget’s diseases of the bone, chronic liver disease, and osteomalacia. All women in this study were at least 1 year from their last menstrual period or had a bilateral oophorectomy. Subjects were stratified into different groups based on serum E2 (pg/ml) as follows: (1) ≤10.5; (2) 10.6–13.9; (3) 14.0–17.4; and (4) ≥17.5.

### Body Mass Index (BMI)

Subjects were requested to empty their pockets, remove shoes, heavy clothing, and heavy jewelries (e.g., watches, necklaces, and bracelets) to measure their body weight using a standard weighing scale. A stadiometer was used to measure height. BMI was calculated as body weight in kilograms divided by the square of the height in meters (kilograms/meters^2^).

### Body Composition

Because body composition was not part of the original protocol in one study and was added on after several women were enrolled in the study (11, 14), we only have the body composition data in 147 women. Total body mass, FFM, FM, and truncal fat were measured using whole body dual-energy X-ray absorptiometry (Hologic Delphi 4500/w; Hologic, Waltham, MA, USA; Enhanced Whole Body 11.2 software version, Hologic) as previously described (15). The coefficients of variation for these measurements in our laboratory are <2% (15). The percentages of total FM were obtained from the estimated readings given by the machine for the different regions of interest. % FFM was calculated as the FFM divided by the whole body mass multiplied by 100 [(FFM/whole body mass) × 100].

### Biochemical Data

Serum E2 levels were measured by ultrasensitive radioimmunoassay (RIA, Diagnostic System Laboratory, Webster, TX, USA) (11, 14). The coefficient of variability for this assay in our laboratory is less than 10% (11, 12, 16).

### Statistical Analysis

Between-group differences in body composition, BMI, age, and E2 were assessed by analysis of covariance. The main outcome investigated, total FM, was normally distributed in our population (*p* = 0.41) according to the Shapiro–Wilk test. We performed power calculation for the definition of the groups to optimize the trade-off between-group sample sizes versus variances; this resulted in 4 groups with a minimum of 32 participants per group with 80% power and 0.05 alpha to detect a difference of at least 8 kg of FM between the nadir (group 3) and the end groups (groups 1 and 4). Differences in body composition and BMI were analyzed adjusting for age. Least significant difference procedure to discriminate among the means was performed as *post hoc* analysis. Data were managed using Excel 2010 (Microsoft, Redmond, WA, USA) and analyzed using Statgraphics X64 (Statgraphics Technologies, The Plains, VA, USA). Results are expressed as the mean ± SD in the text and tables and mean ± SE in the figures. A *p* < 0.05 was considered statistically significant.

## Results

Data from 252 women were included in our analysis (Table 1): mean age (66.1 ± 7.6 years old), weight (79.3 ± 18.5 kg), BMI (29.8 ± 6.5 kg/m^2^), and E2 (14.1 ± 5.6 pg/ml). Subjects’ characteristics according to E2 categories are reported in Table 1.

**Table 1:** Population characteristics according to circulating estradiol (E2).

Groups	1	2	3	4	*p* Value

E2 (pg/ml)	<10.5	10.5–13.9	14.0–17.4	≥17.5	
*N* (%)	53 (21)	87 (35)	56 (22)	56 (22)	
Age (years old)	67.5 ± 7.0	65.9 ± 8.5	65.6 ± 7.8	65.7 ± 7.6	0.52
Body mass index (kg/m^2^)	29.9 ± 6.7*	29.0 ± 6.1*	28.1 ± 5.5*	32.8 ± 6.8	<0.0001
Body weight (kg)	78.4 ± 18.1*	76.0 ± 17.6*	75.8 ± 16.2*	88.9 ± 19.6	<0.001
E2 (pg/ml)	8.4 ± 1.5	12.1 ± 0.8	15.0 ± 0.9	22.0 ± 5.9	<0.001^γ^

*Data are reported as mean ± SD adjusted for age*.

*p < 0.05 for comparison with group 4.

*^γ^All groups differ significantly from each other (*p* < 0.05)*.

Body mass index did not significantly differ between those with and without body composition measurement (29.3 ± 5.9 vs 27.8 ± 5.5 kg/m^2^, respectively, *p* = 0.07). Similarly, E2 levels were not significantly different between the two groups (14.4 ± 6.0 vs 14.1 ± 5.5 pg/ml for those with and without body composition assessment, respectively). However, age and body weight were significantly higher in participants who had body composition (66.6 ± 6.5 vs 63.7 ± 8.6 years old, *p* = 0.002; 78.0 ± 18.0 vs 73.2 ± 16.8 kg, *p* = 0.03, respectively).

**Figure 1:**
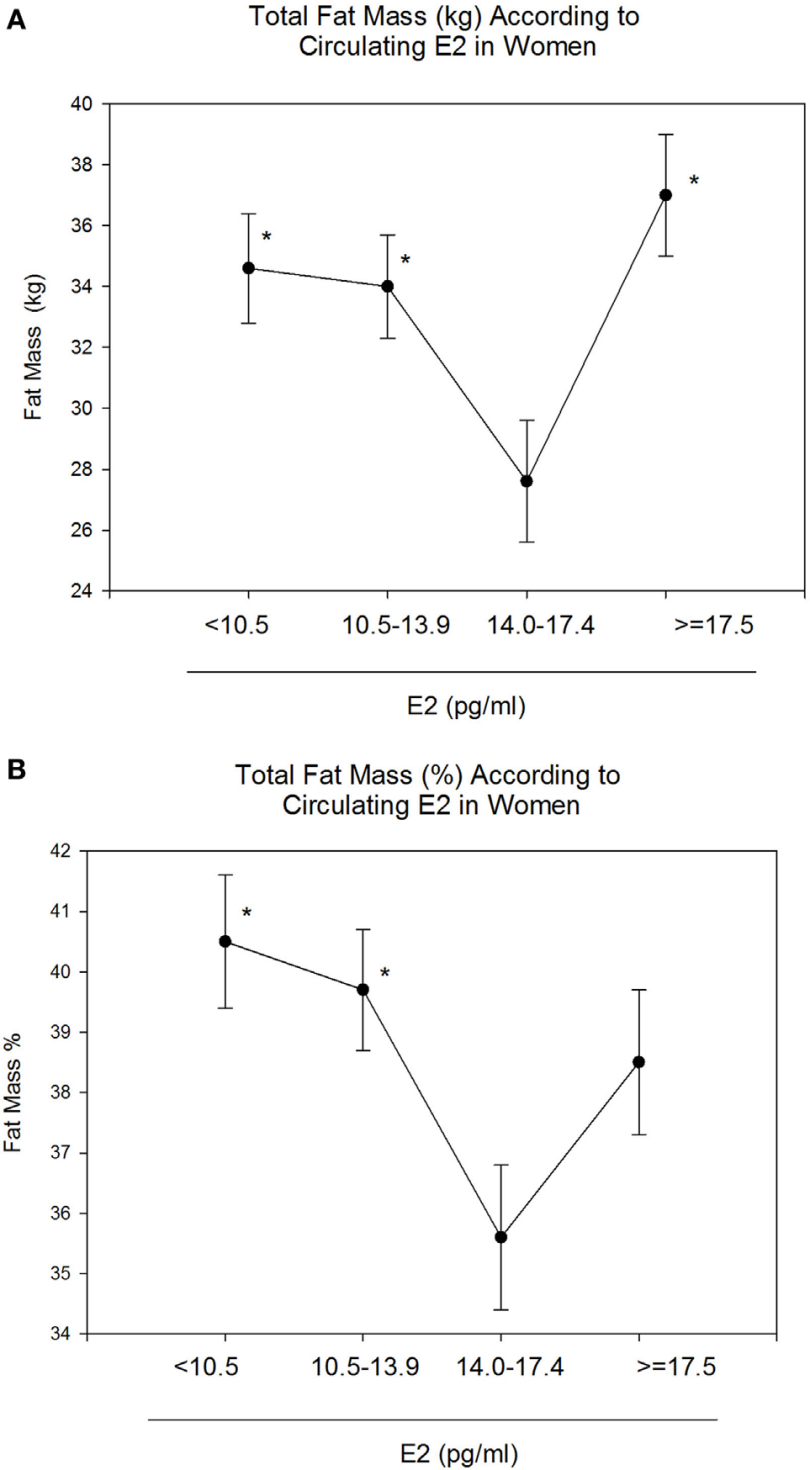
Total fat mass (FM) (kg and %) according to estradiol (E2) categories. E2 levels between 14.0 and 17.4 pg/ml correspond to the lowest total FM [kg–%, **(A,B)**]. Data are reported as mean ±SE. *Comparison with group 3 (E2: 14.0–17.4), *p* < 0.05.

**Figure 2:**
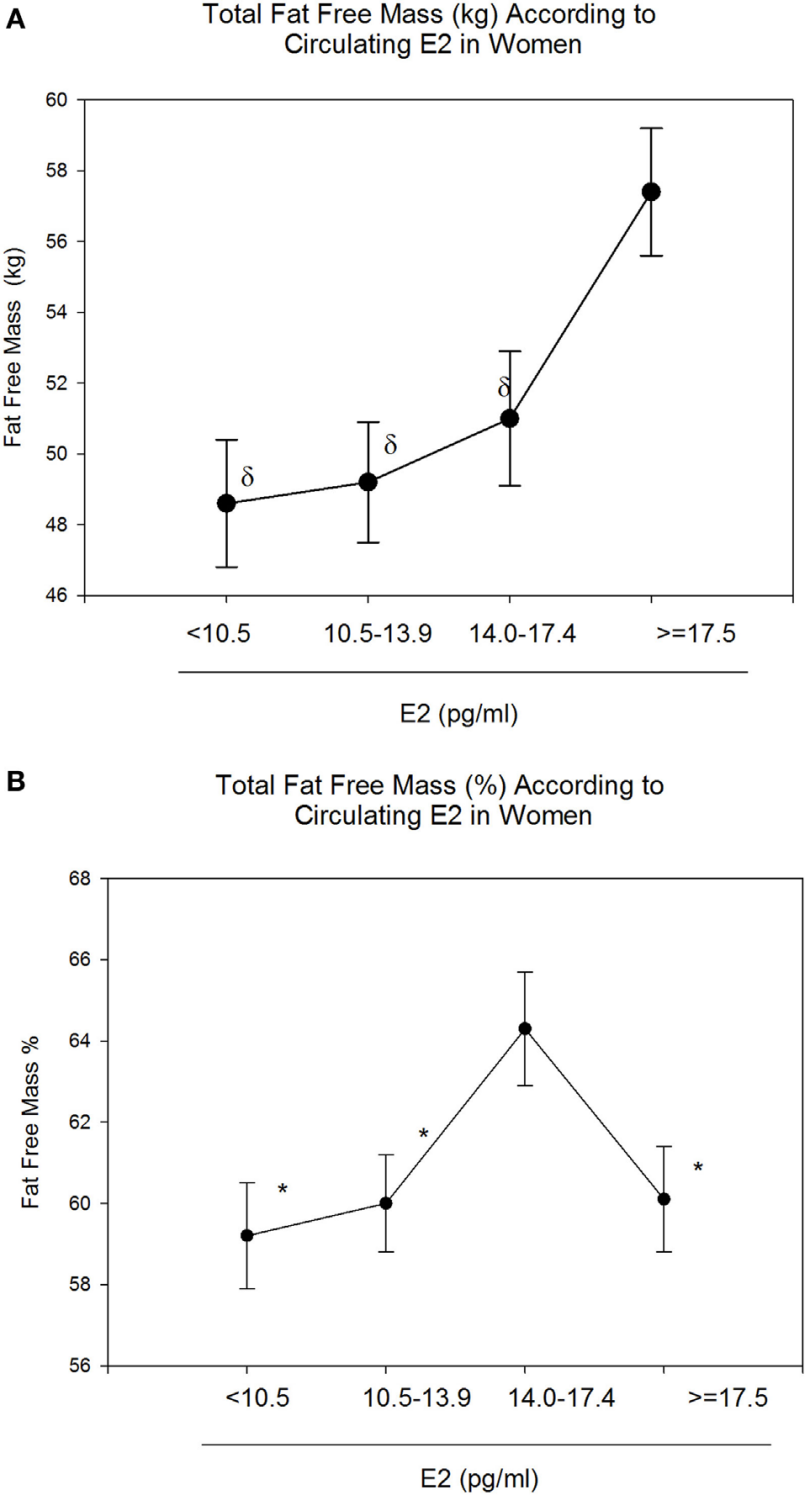
Total fat-free mass (FFM) (kg and %) according to estradiol (E2) categories. E2 levels between 14.0 and 17.4 pg/ml correspond to the highest FFM (%) **(B)**. However, absolute FFM increases with increasing E2 **(A)**. Data are reported as mean ± SE. *Comparison with group 3 (E2: 14.0–17.4), *p* < 0.05; ^δ^Comparison with group 4 (E2: 10.5–13.9), *p* < 0.05.

Body mass index and body weight were the highest in group 4 (E2 ≥ 17.5 pg/ml, Table 1) and were both positively correlated to circulating E2 (*r* = 0.15 and *p* = 0.01 for both variables). On the other hand, total FM (kg) and % FM did not follow a linear relationship with circulating E2 (*r* = 0.07, *p* = 0.34 and *r* = −0.04, *p* = 0.56, respectively). However, analysis according to the different categories of E2 levels showed that both FM (kg) (Figure 1A) and % FM (Figure 1B) follow a U-shaped trend according to E2 levels. FFM (kg) increased with increasing E2 (*r* = 0.22, *p* = 0.007, Figure 2A), while % FFM did not follow a linear relationship with E2 (*r* = −0.001, *p* = 0.98). % FFM in fact follows a bell-shaped curve (Figure 2B) with women in group 3 having the highest % FFM. Truncal FM (kg) was the lowest in group 3 (E2: 14.0–17.4 pg/ml), while % truncal FM and % truncal lean mass did not differ significantly among the groups (Table 2).

**Table 2:** Body composition of the trunk according to estradiol (E2) categories.

Groups	1	2	3	4	*p* Value

E2 (pg/ml)	<10.5	10.5–13.9	14.0–17.4	≥17.5	
*N* (%)	36 (24.5)	42 (28.5)	34 (23)	35 (24)	
Trunk fat mass (FM) (kg)	16.0 ± 6.1^δ^	17.1 ± 7.5*	13.8 ± 5.8^δ^	19.1 ± 6.5*	<0.01
Trunk FM (%)	32.1 ± 6.8	34.4 ± 8.9	32.5 ± 7.9	37.4 ± 7.2	0.16
Trunk lean mass (kg)	21.8 ± 2.3	22.3 ± 6.3	21.9 ± 2.7	24.7 ± 4.6	0.17

*Data are reported as mean ± SD adjusted for age*.

*p < 0.05 for comparison with group 3.

*^δ^p < 0.05 for comparison with group 4*.

## Discussion

Our study showed that postmenopausal women with circulating E2 between 14.0 and 17.4 pg/ml measured by RIA have the best body composition profile with the lowest total and % FM, and the highest % FFM. Furthermore, FM in postmenopausal women followed a U-shaped distribution according to E2 levels, with individuals with E2 levels <14.0 and >17.4 pg/ml having higher body fat.

According to the US Centers for Disease Control and Prevention, obesity and its related disorders are a leading cause of mortality worldwide (1). Hormones play a key role in obesity etiology and progression, though obesity is known to be multifactorial in origin, with causes including genetic, lifestyle, and environmental factors. Gender-dependent adipose tissue distribution and body composition changes after menopause are among the clinical observations linking estrogen to adipocyte development (5). During menopausal transition, women experience a shift from a gynoid to an android fat distribution (5, 7), and become three times more likely to develop obesity and metabolic syndrome than premenopausal women (17). Consistently, visceral FM reduction and improved cardiometabolic profile were reported in a meta-analysis including over 100 clinical trials (18) of women undergoing postmenopausal hormone replacement. The role of E2 in regulating fat distribution is well illustrated in male-to-female transgender patients having a change from an android to a gynoid fat habitus after 12 months of E2 therapy (19).

Interestingly, obesity is often accompanied by a state of relative hyperestrogenemia because of the high aromatase expression/activity in the adipocytes of obese subjects (10). Considering the positive role exerted by estrogens on adipose mass and distribution, the coexistence of high-visceral fat content and elevated estrogen levels in obese individuals may appear inconsistent. This scenario raises the possibility that adipocytes in obese individuals, who have high E2 levels, are not as sensitive to the lipolytic effects of estrogens. This in turn may suggest a different mechanism for the increased body fat at both ends of the estrogen spectrum, from estrogen deficiency on one end to estrogen resistance on the other end. Another explanation for the association between higher body fat at higher end of estrogen level spectrum is the type of obesity developed from a multifactorial cause, independent of estrogen levels. Obesity, together with its associated high aromatase activity, then subsequently leads to the development of higher estrogen levels and possibly to E2 resistance.

To date, results from studies exploring the correlation between estrogens and body fat or BMI are contradictory (20–25). Despite numerous studies investigating the impact of estrogen adequacy or lack thereof on FM (4), to the best of our knowledge, there is no information on the optimum circulating E2 in relation to body fat and FFM in postmenopausal women. Our study showed the existence of a narrow range of serum E2 (14.0–17.4 pg/ml) for which postmenopausal women have the lowest total and % body fat, and highest % FFM. This identifies the E2 levels associated with the best body composition profile in this population. Furthermore, FM followed a U-shaped curve according to E2 levels, with those with E2 levels below 14.0 and above 17.4 pg/ml having higher FM. Considering our results, it is possible that the persistently high-body fat in obese women leads to downregulation of ER-alpha (ERα) in adipocytes and reduced E2 sensitivity despite higher E2 levels.

Estrogen is also the main sex hormone regulating FM in men (26). In fact, estrogen resistance was first described in a man with an inactivating mutation of the ERα (23). Besides the peculiar skeletal profile, the subject was obese (23). Both ERα and ER-beta (ERβ) are expressed in adipose tissue; however, the former seems to be more relevant to its regulation (4). ERα knock-out male and female mice had higher adiposity compared with wild-type mice, while body fat in ERβKO mice was similar to wild-type mice (4). A study conducted on Swedish women reported lower ERα expression in the subcutaneous adipose tissue of obese (*N* = 17) when compared with non-obese (*N* = 16), suggesting a different sensitivity to estrogens depending on BMI (27). On the other hand, ERα expression in subcutaneous adipose tissue was found to increase after weight loss in 23 obese women (28). Since body composition and estrogen levels were not measured in these studies, one can only speculate, but not confirm, that increased body fat and higher estrogen levels were present in these patients. Given our data, it is possible that obese subjects in that study could have either high- or low-circulating E2.

We speculate that a possible sequence of events following the drop in estrogen levels during menopause which include the physiologic increase in body fat, which in turn leads to increased adipose tissue production of estrogen in an effort to compensate for the expanded FM. However, the persistent hyperestrogenemia could lead to ER downregulation and thus reduced adipocyte response to estrogen.

After the age of 40, the prevalence of obesity significantly increases in American women (29) and E2 deficiency is postulated as a critical triggering factor (5). E2 deficiency promotes metabolic dysfunction predisposing to metabolic syndrome, type 2 diabetes, and cardiovascular events (7). Considering the regulation of adipose tissues by estrogen and its implications on the cardiometabolic health (7), the identification of optimum E2 values associated with the best body composition profile would be of clinical relevance. In our group of postmenopausal women, serum E2 values between 14.0 and 17.4 pg/ml corresponded to the best body composition profile; aside from the lowest absolute and percent FM, % FFM was also relatively higher in group 3. FFM increased with increasing E2 levels possibly because of the beneficial effect exerted by E2 on both bone mineral density (30) and muscle homeostasis as suggested by a very recent publication (31).

Our study has limitations. We used of RIA for E2 measurement. Liquid chromatography/mass spectrometry (LC/MS) is considered the gold standard for sex steroid assay because of its high sensitivity and ability to detect very low E2 concentrations (32). However, RIA has been used in prior studies with good reproducibility (30). Nevertheless, according to a study by Hsing et al. exploring the reproducibility of both techniques, steroid hormone levels measured by RIA and MS were highly correlated despite the consistently higher E2 assayed by RIA (32). More importantly, they concluded that the intra- and inter-assay variability of both techniques were equally reliable (32). It is thus possible that the E2 cutoffs we identified will be shifted downwards if our E2 assay was done by LC/MS instead of RIA. Other limitations include the small sample size and the lack of adjustment for confounders, such as additional hormones, diet, and exercise (data not available in our study), which could affect body composition. In addition, the cross-sectional study design does not allow us to establish a causal relationship between the outcomes investigated and E2 levels.

In conclusion, to the best of our knowledge, this is the first study investigating the optimum E2 levels associated with the lowest FM and the best body composition in postmenopausal women. Since FM is comparably high in people with low- and high-serum E2, our observation raises questions regarding E2 sensitivity and the ability of E2 to regulate FM and distribution in women with high E2 levels, i.e., E2 ≥ 17.5 pg/ml. Prospective investigation using LC/MS to measure E2 with a bigger sample size is needed to confirm our observation and to explore the possibility of E2 resistance among individuals with relative hyperestrogenemia.

## Ethics Statement

This study was carried out in accordance with the recommendations of the Washington University School of Medicine institutional review board with written informed consent from all subjects. All subjects gave written informed consent in accordance with the Declaration of Helsinki. The protocol was approved by the Washington University School of Medicine institutional review board.

## Author Contributions

RA-V and DV designed the studies. GC, RC, NN, LA, DV, and RA-V conducted the study and collected the data. GC, CQ, and RA-V analyzed and interpreted the data. GC, RC, NN, LA, CQ, DV, and RV drafted the manuscript. Revising manuscript content and approving the final version: all take responsibility for the manuscript content, the integrity of the data analysis, and approval of the final version of the manuscript.

## Conflict of Interest Statement

The authors declare that the research was conducted in the absence of any commercial or financial relationships that could be construed as a potential conflict of interest.
